# Anatomical distribution of mandibular fractures from severe bicycling accidents: A 12‐year experience from a Norwegian level 1 trauma center

**DOI:** 10.1111/edt.12756

**Published:** 2022-04-28

**Authors:** Mats Døving, Ingar Næss, Pål Galteland, Jon Ramm‐Pettersen, Marius Dalby, Tor Paaske Utheim, Nils Oddvar Skaga, Eirik Helseth, Amer Sehic

**Affiliations:** ^1^ Department of Maxillofacial Surgery Oslo University Hospital Ullevål Oslo Norway; ^2^ Department of Oral Biology, Faculty of Dentistry University of Oslo Oslo Norway; ^3^ Institute of Clinical Medicine, Faculty of Medicine University of Oslo Oslo Norway; ^4^ Department of Neurosurgery Oslo University Hospital Ullevål Oslo Norway; ^5^ Department of Ophtalmology Oslo University Hospital Ullevål Oslo Norway; ^6^ Department of Anaesthesiology, Division of Emergencies and Critical Care Oslo University Hospital Ullevål Oslo Norway; ^7^ Department of Research and Development, Division of Emergencies and Critical Care Oslo University Hospital Ullevål Oslo Norway

**Keywords:** bicycling, head protective devices, maxillofacial injuries, tooth injuries

## Abstract

**Background/Aim:**

The mandible makes up a substantial part of the lower face, and is susceptible to injury. Even in helmeted cyclists, accidents may lead to fractures of the mandible because conventional helmets provide little protection to the lower part of the face. In addition, some studies indicate that helmets may lead to an increased risk of mandibular fractures. Thus, the aim of this study was to examine the anatomic distribution of mandibular fractures in injured cyclists and to assess if helmet use influenced the fracture locations.

**Material and Methods:**

Data from a Norwegian Level 1 trauma center were collected in the Oslo University Hospital Trauma Registry over a 12‐year period. Of 1543 injured cyclists, the electronic patient charts of 62 cyclists with fractures of the mandible were retrospectively evaluated in detail. Demographic data, helmet use, and fracture type were assessed.

**Results:**

Sixty‐two patients (4%) had fractures of the mandible, and women had an increased risk (OR 2.49, 95% CI 1.49–4.16, *p* < .001). The most common fracture site was the mandibular body, followed by the condyle. Isolated mandibular fractures occurred in 45% of the patients and 55% had other concomitant facial fractures. There were 42% of the patients with fractures in multiple sites of the mandible, and 42% had a concomitant dentoalveolar injury. Half of the cyclists were wearing a helmet at the time of the accident and 39% were not. There was no significant difference in fracture distribution between the helmeted and non‐helmeted groups.

**Conclusions:**

Fracture of the mandibular body was the most prevalent mandibular fracture type following bicycle accidents. Women had an increased risk of mandibular fractures compared with men, whereas helmet wearing did not affect the anatomical fracture site.

## 1 INTRODUCTION

Bicycling is associated with several health benefits, including reduced all‐cause mortality, cancer, and cardiovascular risk.^1^, ^2^, ^3^, ^4^ In addition, a shift from motorized travel to active transport such as bicycling, may reduce greenhouse gas emissions.^5^ Despite the benefits, bicycle riders are at risk of injuries due to accidents. Maxillofacial injuries are, together with head injuries, the most common injury type in bicycle accidents after injuries to the extremities.^6^ Several studies have shown the distribution of facial fractures in bicycle‐related accidents, with fractures of the mandible ranging from the least to the most prevalent type.^7^, ^8^, ^9^, ^10^ To the authors' knowledge, however, few studies have reported the anatomical distribution of the different types of mandibular fractures.^7^, ^8^, ^11^, ^12^, ^13^, ^14^, ^15^

Helmet use is associated with a risk reduction of about 50% for head injuries^16^, and two recent meta‐analyses found an overall risk reduction of 21% and 32% for facial fractures.^16^, ^17^ There are, however, diverging results on the effect of injury to the lower part of the face. Some studies have shown that helmet wearing does not affect the risk of mandibular fractures.^10^ On the other hand, helmet use has been associated with both a reduced^17^ and an increased^9^ risk of mandibular fractures. Helmet use has also been found to be associated with an increased risk of dentoalveolar injury,^18^ supporting the hypothesis that helmets increase the risk of injury to the lower face. Since helmets may affect the biomechanics, and consequently, the type of facial fractures sustained in bicycling accidents, the aim of the present study was to examine the anatomical distribution of mandibular fractures in bicycle‐related accidents and to investigate if helmet use influences the location of the fractures.

## 2 MATERIALS AND METHODS

Oslo University Hospital, Ullevål is a regional, Level I trauma center for approximately 3 million people. The study used prospectively collected data from the Oslo University Hospital Trauma Registry (OUH‐TR), a custom built hospital based registry. Eligible for inclusion in the OUH‐TR are all patients admitted with trauma team activation. Furthermore, all patients with penetrating injuries to the head, neck, torso and/or extremities proximal to the elbow or knee, all patients with Injury Severity Score (ISS)^19^ ≥ 10, and patients with AIS Head severity code ≥3 are also included.^20^ The study included patients admitted in the period 2005–2016, whether they were admitted to OUH‐U directly or via a local hospital within 24 h after injury. All injuries were classified according to the Abbreviated Injury Scale 1990 Revision Update 98 (AIS).^21^

Data from all patients admitted with bicycle‐related injuries in the OUH‐TR were obtained. Demographic variables and information on helmet use were acquired. AIS‐codes were examined in order to identify patients with facial fractures and dentoalveolar injuries, and the electronic patient charts of cyclists with fractures of the mandible were thoroughly examined. Information regarding mandibular fracture type and treatment was obtained from a retrospective patient chart review. In addition, fractures of the frontal bone were registered, as they do not have a unique AIS code. The following fracture types were registered: angle, body, condyle, coronoid process, and ramus. Fractures anterior to the angle of the mandible were classified as fractures of the body, and fractures of the condylar head, neck, and sub‐condylar region were grouped together as fractures of the condyle.

Following the review of the electronic patient charts, the patient details were anonymized, and the study was approved by the Data Protection Officer at OUH (17/18831) who considered it exempt from patient consent requirements.

Normally distributed patient characteristics are presented as means with standard deviations (SD) or percentages. Differences in normally distributed continuous variables were calculated using Student *t*‐tests, while either Fisher's exact *t*‐test or Chi‐square test were employed to detect differences in categorical variables. Logistic regression analyses were performed to compare age, gender, ISS, and fracture risk. The results are presented as odds ratios (OR) with 95% confidence intervals (CI). Statistical analyses were conducted using IBM SPSS version 25 for Windows (SPSS, Inc.) and Stata (StataCorp. 2021. *Stata Statistical Software: Release 17*. College Station, Tx: StataCorp LLC). A two‐sided *p* < .05 was considered to be statistically significant.

## 3 RESULTS

A total of 1,570 patients with bicycle‐related accidents were admitted during the study period, of whom 27 patients were pedestrians who had been struck by a cyclist and they were excluded accordingly. Of the remaining 1,543 patients, 66 were registered with fractures of the mandible. Among these, four patients were excluded after reviewing the electronic patient charts which revealed an incorrect diagnosis. Consequently, 62 patients (4%) with 100 mandibular fractures were included in the study.

**FIGURE 1:**
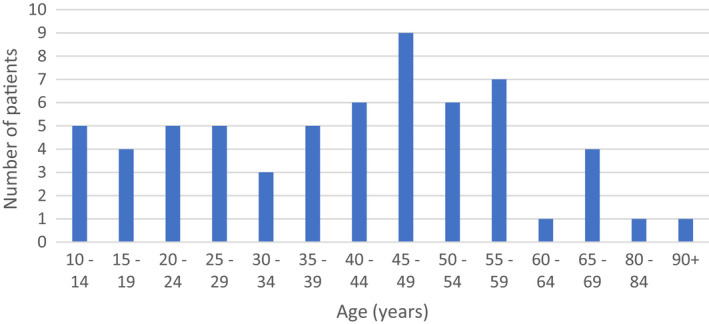
Age distribution of the cyclists with mandibular fractures (*n* = 62)

The age‐distribution of the patients is presented in Figure 1. The mean age of the patients who sustained fractures of the mandible was 40.9 years (SD 17.9) (Table 1). Age did not affect the risk of mandibular fractures (OR 1.01, 95% CI 0.99–1.02, *p* = .472). Twenty‐nine (47%) patients were women, and 33 (53%) were men. Women showed a higher risk of mandibular fractures compared to men (crude OR 2.48, 95% CI 1.48–4.13, *p* < .001; adjusted for age OR 2.49, 95% CI 1.49–4.16, *p* < .001). The most common mechanism of injury was a single bicycle accident (*n* = 49, 79%) followed by collision with a motor vehicle (*n* = 13, 21%) (Table 1). As for injury severity, there was an association between mandibular fractures and increasing ISS (crude OR 1.03, 95% CI 1.02–1.05, *p* < .001; adjusted for age and gender OR 1.03, 95% CI 1.02–1.05, *p* < .001).

**TABLE 1:** Demographic characteristics of the patients with and without mandibular fractures

	Total (*N* = 1543)	Mandibular fracture (*N* = 62)	No mandibular fracture (*N* = 1481)	*p* Value
Age (years), mean (SD)	39.2 (18.9)	40.9 (17.9)	39.2 (18.9)	.472^a^
Gender				
Female, *n* (%)	417 (27)	29 (47)	388 (26)	<.001^b^ ^,*^
Male, *n* (%)	1126 (73)	33 (53)	1093 (74)	
Time of accident				
Winter, *n* (%)	38 (2)	1 (2)	37 (2)	.953^b^
Spring, *n* (%)	400 (26)	16 (26)	384 (26)	
Summer *n* (%)	758 (49)	32 (51)	726 (49)	
Fall *n* (%)	347 (23)	13 (21)	334 (23)	
Type of accident				
Single bicycle crash, *n* (%)	1053 (68)	49 (79)	1004 (68)	.321^c^
Collision with a motor vehicle, *n* (%)	410 (27)	13 (21)	397 (27)	
Collision with another cyclist, *n* (%)	63 (4)	0	63 (4)	
Collision with a pedestrian	7 (0)	0	7 (0)	
Other	10 (1)	0	10 (1)	
Alcohol				
Yes, *n* (%)	144 (9)	6 (10)	138 (9)	.401^b^
No, *n* (%)	119 (8)	2 (3)	117 (9)	
Not tested, *n* (%)	1280 (83)	54 (87)	1226 (82)	
GCS				
15, *n* (%)	1146 (74)	46 (74)	1100 (74)	.589^b^
14–12, *n* (%)	221 (14)	7 (11.5)	214 (15)	
11–9, *n* (%)	63 (4)	2 (3)	61 (4)	
≤8, *n* (%)	113 (8)	7 (11.5)	106 (7)	
ISS				
≤8, *n* (%)	583 (38)	11 (18)	552 (37)	.02^b^ ^,*^
9–14	454 (29)	24 (38)	430 (28)	
15–24	319 (20)	16 (26)	303 (20)	
≥25	207 (13)	11 (18)	196 (13)	

Abbreviations: GCS; Glascow coma scale; ISS; injury severity score; SD; standard deviation.

^a^ Independent sample Student *t*‐test.

^b^ Chi square test.

^c^ Fisher's exact test.

^*^ *p* < .05.

**FIGURE 2:**
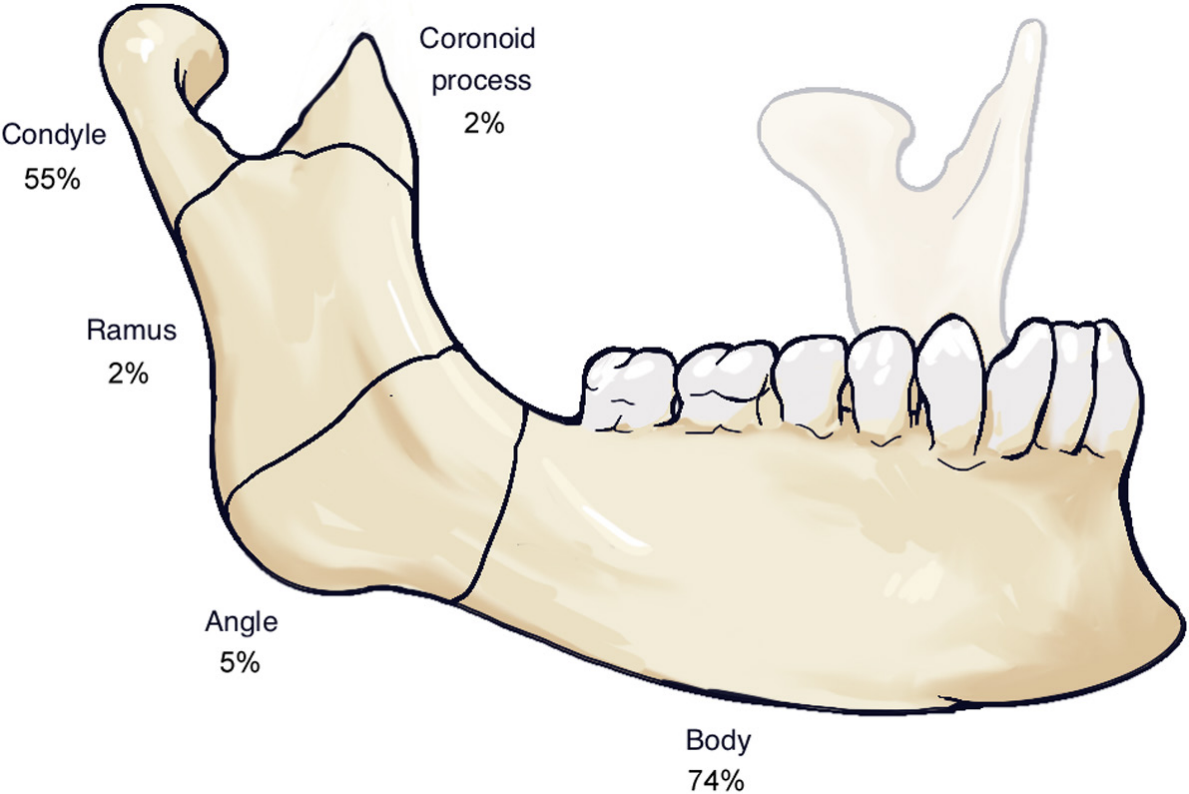
Distribution of anatomic fracture location in the 62 cyclists with fractures of the mandible

The most common fracture location was the mandibular body, which was fractured in 46 patients (74%). Fractures of the condyle occurred in 34 patients (55%) and of these, 13 patients (21%) had bilateral fractures. The distribution of the fracture types is presented in Figure 2.

**FIGURE 3:**
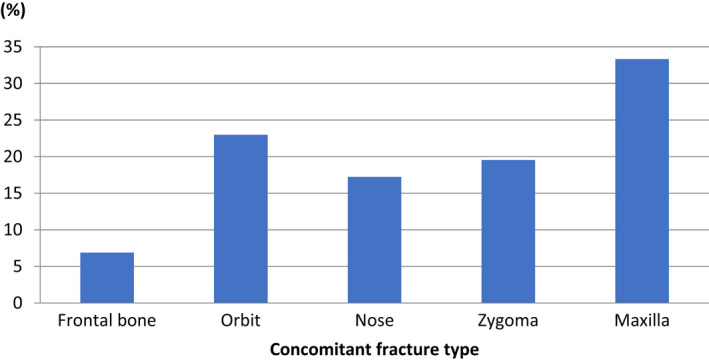
Occurence of concomitant facial fractures in the 62 cyclists with mandibular fractures

There were 35 patients (56%) who had fractured one anatomical site of the mandible, 16 (26%) had fractures in two locations, and 11 patients (18%) had triple fractures of the mandible. Furthermore, isolated fractures of the mandible occurred in 28 (45%) patients, whereas 34 (55%) patients had concomitant facial fractures. The occurrence of concomitant facial fractures is presented in Figure 3. Patients with concomitant facial fractures had a higher mean age compared with those who only had fractures of the mandible (45.7 (SD 18.3) years vs. 35.1 (SD 16.0) years, *p* = .02).

Dentoalveolar injuries were present in 26 (42%) patients with mandibular fractures and the distribution was dental fracture; 12 (19%), avulsion; 6 (10%), alveolar process fracture; 5 (8%), luxation; 3 (5%), and unspecified dental injury 3 (5%). There was no difference in mean age between patients who had concomitant dentoalveolar injuries and those who did not (44 years (SD 17.79) vs. 39 years (SD 18.00), *p* = .28). An association was found between the combination of triple mandibular fractures and dentoalveolar injuries (single; OR 0.74, 95% CI 0.24–2.33, *p* = .57, double; OR 0.26, 95% CI 0.04–1.16, *p* = .071, triple; OR 9.00, 95% CI 1.55–91.21, *p* = .005).

Half of the cyclists (*n* = 31) who sustained fractures of the mandible were wearing a helmet at the time of the accident while 24 (39%) were not. Helmet status was not registered in seven (11%) patients. There was no association between helmet wearing and the mandibular fracture types when analysed in isolation (Table 2).

**TABLE 2:** Distribution of 100 mandibular fractures and helmet use in 62 patients

Fracture type	Total fractures (*n* = 100)	Unknown helmet status (*n* = 7)	Helmeted (*n* = 31)	Non‐helmeted (*n* = 24)	OR (95% CI)^a^
Body, *n* (%)	48 (48)	6 (86)	25 (81)	15 (63)	2.5 (0.74–8.43)
Condyle, *n* (%)	46 (46)	4 (57)	15 (16)	15 (63)	0.56 (0.19–1.67)
Angle, *n* (%)	3 (3)	1 (14)	1 (3)	1 (4)	0.77 (0.05–12.92)
Ramus, *n* (%)	1 (1)	0	0	1 (4)	0.37 (0.01–11.54)
Coronoid process, *n* (%)	1 (1)	0	1 (3)	0	0.37 (0.01–11.54)

Abbreviations: CI, confidence interval; OR, odds ratio; SD, standard deviation.

^a^ Cyclists with unknown status for helmet use not included in the analysis.

Most of the patients (42%) with fractures of the mandible underwent open reduction and internal fixation (ORIF). Seventeen patients (27%) were treated with intermaxillary fixation (IMF), either as the sole treatment or in conjunction with ORIF, and 16 (25%) patients were treated conservatively without operative treatment or IMF. Patients with fractures of the mandibular body more often underwent ORIF than patients with other fracture types (OR 3.64, 95% CI 1.11–11.94, *p* = .028). There was a positive association between IMF and triple mandibular fractures (OR 12.44, 95% CI 2.75–56.31, *p* < .001) and unilateral fractures of the condyle (OR 20.25, 95% CI 4.38–91.28), *p* < .001). There was also an association between mean age in those with or without IMF (mean age 33.2 (SD 16.8) vs. 43.8 (SD 17.6), *p* = .036). There was no difference in mean age for those who had and those who did not have conservative treatment (42.6 (SD 18.8) vs. 40.4 (SD 17.8), *p* = .674), or ORIF (42.5 (SD 19.0) vs. 37.8 (SD 15.8), *p* = .333). In addition, there was no difference in treatment type for helmeted vs. non‐helmeted cyclists—that is, conservative (40% vs. 62.5%, *p* = .134), IMF (56.3% vs. 56.4%, *p* = .991) or ORIF (55.6% vs. 57.9%, *p* = .868).

## 4 DISCUSSION

The scientific evidence of the distribution of mandibular fractures in bicycling accidents is limited. Therefore, the aim of the present study was to investigate the anatomical injury patterns of mandibular fractures in cyclists admitted to a Level 1 trauma center. Furthermore, the study sought to examine the association between mandibular fracture types and helmet use, concomitant facial fractures and dentoalveolar injuries, as well as the choice of treatment.

The body of the mandible was the most frequent fracture location in bicycle‐related mandibular fractures in the present study. This is in accordance with the findings of Lin et al. in their study of bicycling injuries from a Level I trauma center in Taiwan^11^ as well as a recent Japanese study on road traffic accidents.^15^ In the latter, bicycling accidents accounted for around two thirds of the maxillofacial injuries. However, that study did not report the specific fracture pattern of the mandibular fractures for the eighteen cyclists included.^15^ The authors did, nevertheless, report a similar percentage of single mandibular fractures as in the present study (58%), and that fractures of the condyle were the second most frequent fracture location.^15^ This is in contrast to other studies where fractures of the condyle were more common.^7^, ^8^, ^12^, ^13^, ^14^

The direction and the amount of force during an injury influence the fracture site of the mandible,^22^, ^23^ and several authors have ascribed the higher risk of condylar fractures to trauma applied to the symphyseal region with consequent indirect fracture of the condyle.^11^, ^12^, ^14^ Thus, it is possible that a greater force applied to the symphyseal region may lead to fracture at the site of the blow rather than at the condylar area. This is supported by a recent study which performed a finite element analysis of bicycling accidents and fractures of the mandible.^24^ In contrast to previous studies which assessed bicycle‐related fracture patterns of the mandible, the current study exclusively included patients examined by a trauma team and/or patients with either confirmed or high likelihood of serious injury. Therefore, the difference in fracture distribution could be due to more severe accidents in this study's population. This is consistent with the present study's finding of a positive association between fractures of the mandible and increasing ISS. Another reason for the observed difference could be the heterogeneity between the study populations, such as age or gender. Although age has been identified as a risk factor for maxillofacial fractures,^24^ the present study found no association between age and fractures of the mandible. The patients who suffered combinations of fractures of the mandible and other regions of the maxillofacial skeleton, nevertheless had a higher mean age compared with those who only had mandibular fractures. This study also found that women had an increased risk of sustaining fractures of the mandible, which may be due to gender differences in shape or bone structure, with men having bigger, and possibly more robust mandibles.^23^, ^25^, ^26^, ^27^ Furthermore, women undergo a more pronounced decline of mandibular bone quality with age compared with men.^28^ The observed difference in fracture risk could also be the result of different riding styles or other fundamental physiological differences between genders.^29^, ^30^

Although helmets provide protection of the head and upper part of the face, they may increase the risk of fractures of the mandible if it leads to a second blow to the lower part of the face after the helmet has hit an object.^31^ Consequently, it is possible that helmet use can alter the anatomical distribution of mandibular fractures. However, the present study found no difference in anatomical fracture distribution of the mandible when comparing helmeted and non‐helmeted cyclists. To the best of the authors' knowledge, this has only been examined in one previous study, which was limited by a small sample size of only seven patients.^11^ Although the present study included more patients, it is also limited by size. Thus, larger studies are warranted to further examine the effect of helmet wearing on injury to the lower face.

Full‐face helmets may provide better protection of the lower face compared with open helmets, and although full‐face helmets are more common in motorcycling and downhill cycling, they are rarely seen in regular cyclists. The reason for this could be that they are not available through retail stores or because they are considered less fashionable or impractical due to their larger size. However, new technology for helmet design, such as a self‐inflating helmet which includes protection of the lower face is already commercially available and may become more common in the future. Nevertheless, more research is needed to improve helmet design. This is evident by a recent meta‐analysis which found no difference in the occurrence of facial fractures between full‐face and open helmets in motorcyclists.^32^

Yamamoto et al.^12^ found 22 mandibular alveolar fracture lines in 175 patients with mandibular fractures caused by bicycling accidents but the study did not report information on other types of dental injuries. In the present study, a high proportion of the cyclists with fractures of the mandible had concomitant dentoalveolar injuries (42%), of which tooth fractures were the most frequent type. Patients with triple fractures of the mandible had an increased risk of dentoalveolar injury which is probably because a force high enough to produce fractures in three different anatomical regions is more likely to also cause accompanying injuries to the teeth.

The current study has some limitations. The study only included patients admitted to a trauma center due to serious or potentially serious injury. Thus, cyclists involved in less serious trauma could have a different prevalence and distribution of mandibular injuries. For instance, it is possible that open fractures of the mandibular body were considered more serious than closed fractures of the condyle, and that the former were referred to the trauma center but not the latter. The same could be true for patients with combinations of facial fractures and dentoalveolar injuries which may appear more serious than isolated fractures of the face. The generalizablity of this study is therefore mostly relevant for patients with serious bicycle‐related injuries. Furthermore, it is possible that some cyclists were fully protected by their helmet and consequently did not sustain any injury. This may have underestimated the protective effect of helmets.

The even distribution of patients in the helmeted and non‐helmeted group is a strength of the current study. However, helmet‐status was not registered in 11% of the patients which may have skewed the results. Another strength of the study is the sole inclusion of patients from a dedicated trauma center and that the electronic patient charts were thoroughly re‐examined by a single researcher.

## 5 CONCLUSIONS

Fractures of the body, followed by fractures of the condyle, were the most common mandibular fracture types in bicycle‐related accidents. There was no association between mandibular fracture type and helmet use. Females had an increased risk of sustaining fractures of the mandible compared with men. Additional studies are warranted to further understand the role of conventional bicycle helmets for injuries to the mandible and the lower face.

## CONFLICT OF INTEREST

The authors have no conflict of interest to declare.

## AUTHOR CONTRIBUTION

Mats Døving involved in conception and study design, patient chart review, analysis and interpretation of data, and drafting and revision of the manuscript. Ingar Næss performed analysis and interpretation of data and revision of the manuscript. Pål Galteland involved in data interpretation and revision of the manuscript. Jon Ramm‐Pettersen and Marius Dalby performed study design, analysis and interpretation of data and revision of the manuscript. Tor Paaske Utheim, Nils Oddvar Skaga, and Eirik Helseth performed data interpretation and revision of the manuscript. Amer Sehic involved in study design, data interpretation, and revision of the manuscript.

## Data Availability

Research data are not shared.
